# Intestinal Cgi-58 Deficiency Reduces Postprandial Lipid Absorption

**DOI:** 10.1371/journal.pone.0091652

**Published:** 2014-03-11

**Authors:** Ping Xie, Feng Guo, Yinyan Ma, Hongling Zhu, Freddy Wang, Bingzhong Xue, Hang Shi, Jian Yang, Liqing Yu

**Affiliations:** 1 Department Of Biochemistry, Wake Forest University Health Sciences, Winston-Salem, North Carolina, United States of America; 2 Institute Of Medicinal Plant Development, Chinese Academy Of Medical Sciences & Peking Union Medical College, Beijing, China; 3 Department Of Animal And Avian Sciences, University Of Maryland, College Park, Maryland, United States of America; 4 Department Of Biology, Georgia State University, Atlanta, Georgia, United States of America; 5 Department Of Physiology, University Of South Alabama College Of Medicine, Mobile, Alabama, United States of America; Wayne State University, United States of America

## Abstract

Comparative Gene Identification-58 (CGI-58), a lipid droplet (LD)-associated protein, promotes intracellular triglyceride (TG) hydrolysis in vitro. Mutations in human CGI-58 cause TG accumulation in numerous tissues including intestine. Enterocytes are thought not to store TG-rich LDs, but a fatty meal does induce temporary cytosolic accumulation of LDs. Accumulated LDs are eventually cleared out, implying existence of TG hydrolytic machinery in enterocytes. However, identities of proteins responsible for LD-TG hydrolysis remain unknown. Here we report that intestine-specific inactivation of CGI-58 in mice significantly reduces postprandial plasma TG concentrations and intestinal TG hydrolase activity, which is associated with a 4-fold increase in intestinal TG content and large cytosolic LD accumulation in absorptive enterocytes during the fasting state. Intestine-specific CGI-58 knockout mice also display mild yet significant decreases in intestinal fatty acid absorption and oxidation. Surprisingly, inactivation of CGI-58 in intestine significantly raises plasma and intestinal cholesterol, and reduces hepatic cholesterol, without altering intestinal cholesterol absorption and fecal neutral sterol excretion. In conclusion, intestinal CGI-58 is required for efficient postprandial lipoprotein-TG secretion and for maintaining hepatic and plasma lipid homeostasis. Our animal model will serve as a valuable tool to further define how intestinal fat metabolism influences the pathogenesis of metabolic disorders, such as obesity and type 2 diabetes.

## Introduction

In mammals, dietary fats, mainly triglycerides (TG) (triacylglycerols, or TAGs), are emulsified with the help of bile salts. The emulsified lipids are then digested into free fatty acids (FFAs) and monoacylglycerols (MAGs) by secreted pancreatic lipases in the lumen of small intestine. These lipid digestion products, together with bile salts and phospholipids, form mixed micelles, which travel across the unstirred water layer to the brush-border membrane of small intestine and deliver lipid molecules for uptake by absorptive enterocytes [1]. Once inside the enterocytes, FFAs and MAGs are directly carried to the endoplasmic reticulum (ER) for reesterification into TG. Recent studies suggest that re-synthesized TGs in the enterocytes have at least two fates. While the majority of them are transferred to apolipoprotein B48 for assembly into the core of chylomicron (CM) particles in a microsomal triglyceride transfer protein (MTP)-dependent manner, the rest are temporarily stored in the cytosolic LDs. These TG-rich cytosolic LDs have been observed, especially after ingestion of a high-fat diet (HFD), in the enterocytes of mice [2], [3], rabbits [4] and humans [5]. TGs stored in the cytosolic LDs are eventually hydrolyzed for oxidation, or for reesterification in the ER for CM-mediated secretion into lymphatic system [3]. However, the identities of proteins responsible for hydrolysis of cytosolic LD-associated TG in lipoprotein-producing enterocytes are unknown.

One protein implicated in cytosolic TG hydrolysis is Comparative Gene Identification-58 (CGI-58), the fifth member of α/β-hydrolase domain-containing (ABHD) protein family (ABHD5) [6]. Mutations in human CGI-58 cause Chanarin-Dorfman Syndrome (CDS) [7], an autosomal recessive genetic disease characterized by massive accumulation of TGs in the cytosolic LDs in most cell types including absorptive enterocytes of small intestine [8], [9]. CGI-58 was identified as a LD-associated protein [10]–[12] and has been shown to interact with perilipins [10], [11], [13], [14] to promote fat lipolysis. Despite lacking intrinsic TG hydrolase activity, CGI-58 has been shown to function as a cofactor to activate in vitro TG hydrolase activity of adipose triglyceride lipase (ATGL) [15] whose mutations also cause a neutral lipid storage disease in humans [16]. Interestingly, three CDS patients were reported to have recurrent steatorrhea (indicative of fat malabsorption) with no specific cause noted [17], [18]. In a 41-year-old man with CDS, the stool fat output was increased 4-fold [17]. Fecal fat excretion may be increased in other CDS patients, but was not examined or reported. These data imply that CGI-58 may play an important role in fat absorption in humans, but direct experimental evidence in vivo is lacking. We hypothesized that CGI-58 facilitates fat absorption, at least in the postprandial state, by promoting TG hydrolysis in cytosolic LDs.

Mice with whole-body deficiency of CGI-58 die shortly after birth due to a permeability barrier defect of the skin [19], making it difficult to assess the role of CGI-58 in lipid absorption in enterocytes. Here we report that selective inactivation of *CGI-58* by Cre-mediated recombination in the mouse small intestine leads to massive accumulation of LDs in enterocytes, which is associated with a significant reduction in postprandial lipid absorption and alterations in systemic lipid homeostasis.

## Experimental Procedures

### Generation of Intestine-specific CGI-58 Knockout Mice

The creation of the mice with CGI-58 alleles flanked by two *LoxP* sites (CGI-58-floxed mice) was descibed in detail previously [20]. Intestine-specific CGI-58 knockout mice were generated by mating heterozygous CGI-58-floxed (CGI-58^f/+^) mice with B6.SJL-Tg(Vil-cre)977Gum/J mice (Jackson Laboratory, Stock #004586), followed by crossing CGI-58^f/+^/Vil-cre mice with CGI-58^f/+^ mice to get homozygous CGI-58-floxed mice with Vil-cre transgene, designated CGI-58^f/f/Cre^ or intestine-specific CGI-58 knockout, and homozygous CGI-58 floxed mice without Vil-cre transgene, designated CGI-58^f/f^ or Control. CGI-58^f/f/Cre^ and CGI-58^f/f^ were then crossed to generate their offspring for all experiments.

Mice were housed in a specific pathogen-free animal facility in plastic cages at 22°C with a 12-h light/dark cycle from 6 AM to 6 PM and fed a standard chow diet (Prolab RMH 3000; LabDiet, Brentwood, MO) ad libitum, unless stated otherwise.

### Ethics Statement

All animal procedures were approved by the Institutional Animal Care and Use Committee at Wake Forest University Health Sciences, Winston-Salem, North Carolina, USA, and by the Institutional Animal Care and Use Committee at University of Maryland, College Park, Maryland, USA.

### Diet Studies

Beginning at 5 weeks of age mice were switched from the normal chow diet to a synthetic Western-type high fat diet (HFD) as we have described previously [21]. This synthetic diet contains 40% energy from lard [20.68% (w/w) lard] and 0.2% (w/w) cholesterol. After 6-week-HFD feeding, mice were fasted 4 h and sacrificed by isoflurane inhalation, and tissues were dissected and snap-frozen in liquid nitrogen immediately after collection of blood samples via vena cava. Tissues were stored at −80°C for further analysis.

### Analysis of Serum Parameters and Tissue Lipids

Necropsy plasma samples (n = 8–12) were collected following a 4 h fast during the light cycle in a tube containing 10 µl of a mixture that is composed of 5% EDTA in Tris-HCl (pH 8.0) and 5% NaN_3_ in water. The blood was centrifuged at 12,000×g for 10 min at 4°C. Plasma total cholesterol (TC), free cholesterol (FC), and triglyceride (TG) concentrations were determined by enzymatic assay kits from Pointe Scientific, Inc. (Cat.# C7510-120), Wako (Cat.# 435-35801), and Sigma (Cat.# T2449), respectively, as previously described [22]. The amount of cholesterol ester was calculated by subtracting free cholesterol from total cholesterol and multiplied by 1.67 to convert to cholesterol ester mass. Plasma β-hydroxybutyrate and non-esterified (free) fatty acids levels were measured using commercially available enzymatic reagents (Cat.# 2440-058, Stanbio Laboratory, Boerne, TX; HR Series NEFA-HR(2), Wako Diagnostics, Richmond, VA). Lipids were extracted from ∼80 mg of tissue biopsies and measured enzymatically as described previously [23].

### Determination of Plasma Lipoprotein-cholesterol Profile

An equal amount of plasma sample from each mouse in each group was pooled. The pooled sample was analyzed for the plasma lipoprotein-cholesterol profile by the fast phase liquid chromatography (FPLC) method using a Superose 6 10/300 GL column (GE Healthcare) and a LaChrom Elite HPLC system (Hitachi High Technologies). Briefly, a 50 µl pooled plasma sample from each group was diluted with PBS (0.05 M phosphate, 0.9% sodium chloride, 0.01% EDTA, and 0.01% sodium azide) to 400 µl total volume and then injected onto the FPLC system with online mixing of enzymatic reagents (Cholesterol Liquid Reagent Set, Pointe Scientific, Inc.) with effluent from the column at a flow rate of 0.4 ml/min. The lipoprotein-cholesterol distribution was monitored by a computer.

### Measurement of Intestinal Fat Absorption

After 6 weeks of HFD feeding, mice were separated, individually housed, and fed for 6 days on a test diet. The test diet composition is similar to the HFD except 5% of fat was replaced by the non-absorbable marker sucrose poly-behenate (SPB) [24]. The test diet was prepared by the Diet Core in the primate center of Wake Forest University Health Sciences. In the last 3 days of test diet feeding, fecal samples were collected. The fatty acid content and composition in both the diet and feces were determined by gas chromatography and the fractional absorption of total and individual fatty acids was calculated as described previously [24].

### Postprandial TG Secretion

For analysis of postprandial TG secretion, mice were maintained on HFD for 6 weeks. After a 16 h fast, mice were weighed and administered with 500 mg/kg Tyloxapol (Triton WR-1339, Sigma) via intraperitoneal cavity to block lipoprotein lipase activity. Thirty minutes later, an intragastric bolus of lipids (0.5 ml of olive oil) was administered and blood samples were collected from tail vein at 1, 2, 3, and 4 h after gavage. Plasma TG concentrations were assayed by Wako L-Type Triglyceride M kit (Cat.# 461-08992, Wako Chemical USA, Richmond, VA).

### Measurement of Intestinal TG Hydrolase Activity

The small intestine was collected and divided into 5 equal segments. The second proximal segment of the small intestine (*n = *5) was washed in ice-cold PBS containing 2 U/ml heparin and 1 mM EDTA. The TG hydrolase activity was measured in the whole tissue homogenate exactly as we have described previously using freshly isolated LDs containing radio-labeled TG as substrate [25].

### Intestinal Fatty Acid Oxidation

Fatty acid oxidation activity was measured as we have previously described [25]. After 6 weeks of HFD feeding, mice were fasted for 4 h. The second segment of freshly collected small intestine (200 mg) was homogenized in 600 µl of 0.25 M sucrose containing 1 mM EDTA. The tissue homogenate (1 mg protein) was incubated for 30 min at 25°C in a buffer containing 150 mM KCl, 10 mM HEPES (pH 7.2), 0.1 mM EDTA, 1 mM potassium phosphate buffer (pH 7.2), 5 mM malonate, 10 mM MgCl_2_, 1 mM carnitine, 0.15% fatty acid free-BSA, 5 mM ATP, and 50 µM palmitic acid containing 1 µCi of [9, 10-^3^H(N)] palmitic acid (32.4 Ci/mmol; PerkinElmer, Boston, MA). Reactions were stopped by addition of 200 µL 0.6 N perchloric acid. After removal of unreacted fatty acids by hexane extraction, acid-soluble radiolabeled degradation products in the aqueous phase were measured by liquid scintillation counting and the rates of fatty acid oxidation were presented as pmol/min/mg tissue protein.

### Cholesterol Absorption and Fecal Neutral Sterol Excretion

The fractional cholesterol absorption was determined with oral dual-isotope ratio method as we have described previously [23]. Each of the individually housed mice was given an oral gavage of 100 µl soybean oil containing 0.2 µCi [^3^H]-sitostanol and 0.1 µCi [^14^C]-cholesterol. Mice were returned to their cages and allowed free access to food and water. After 72 h, feces were collected and homogenized in 95% ethanol. A total of 3 ml of homogenized fecal sample was saponified by adding 300 µl of 50% KOH in water and heating at 65°C for 2 h. The lipids were extracted by adding 3 ml of hexane and 3 ml of H_2_O. The radioactivity in the extract was measured by scintillation counting and the ratio of [^14^C]-cholesterol to [^3^H]-sitostanol was then calculated.

Fecal neutral sterol excretion was determined as described previously [23]. Briefly, feces were collected and dried in a 70°C vacuum oven, weighed, and crushed. Approximately 100 mg of feces were placed into a glass tube containing 100 µg of 5α-cholestane as an internal standard. Feces were saponified and the lipids were extracted with hexane. Then the neutral sterol was measured by gas-liquid chromatography. The fecal neutral sterol mass represents the sum of cholesterol, coprostanol, and cholestanone in each sample. Fecal neutral sterol excretion was expressed as µmol sterol/day/100 g body weight.

### Statistical Analysis

All data are presented as Mean ± Standard Error of the Mean (SEM). Statistical analysis was performed by two-tailed Student’s *t* test. Statistical differences with *P*<0.05 were considered significant.

## Results

### Intestine-specific Deletion of CGI-58 in Mice

**Figure 1:**
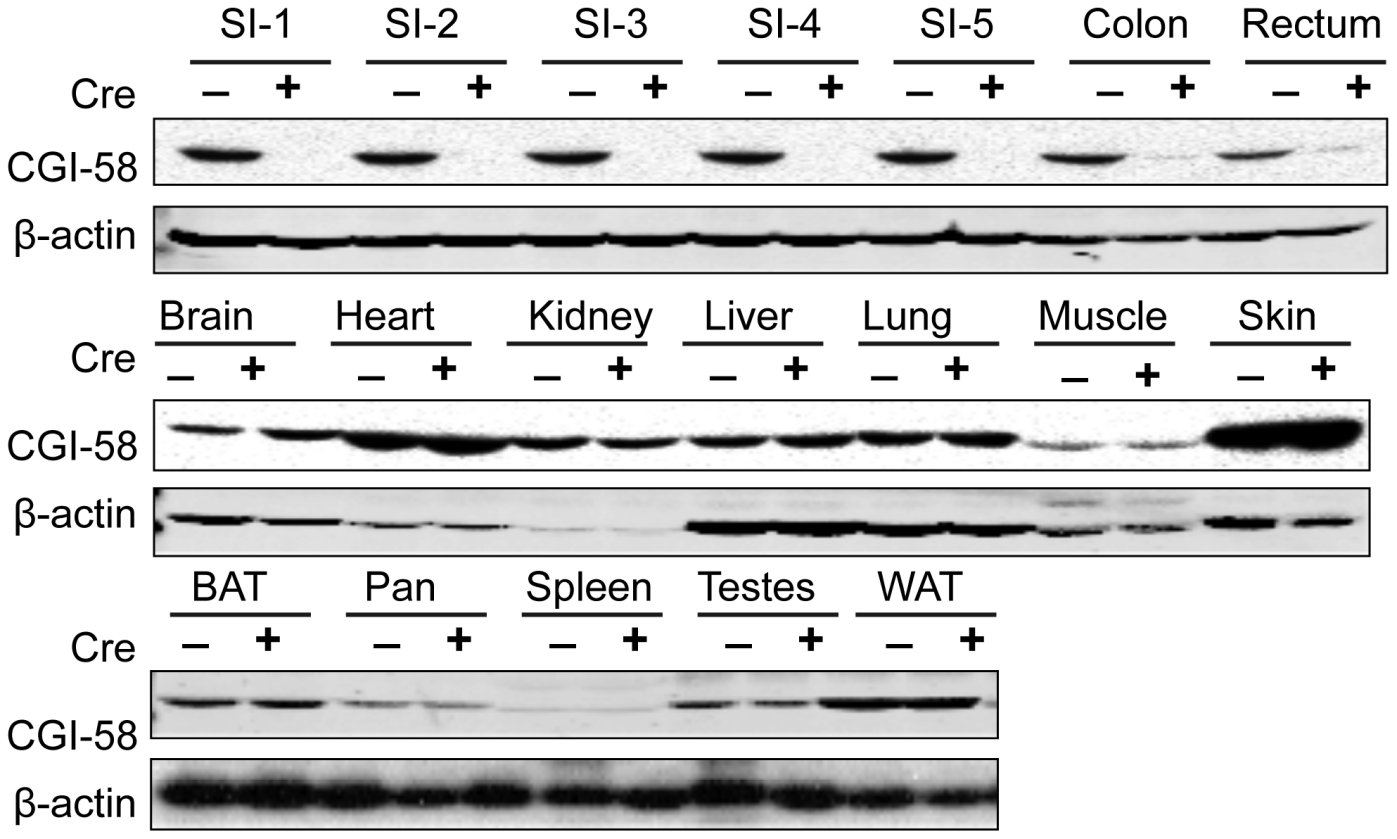
Generation of intestine-specific CGI-58 knockout mice. Immunoblot analysis of CGI-58 protein in intestine-specific CGI-58 knockout mouse model. Tissues were collected from CGI-58^f/f/Cre^ (Cre+) and CGI-58^f/f/^ (Cre−) mice, and homogenized in RIPA buffer. The tissue homogenate (25 µg proteins) was separated by electrophoresis on 4–12% SDS-polyacrylamide gel, transferred to polyvinylidene difluoride membrane, blotted with anti-CGI-58 mouse monoclonal or anti-β-actin antibody as previously described [28]. SI-1 to SI-5, proximal to distal segments of small intestine that was divided into 5 equal segments; BAT, brown adipose tissue; Pan, pancreas; WAT, white adipose tissue.

To confirm the intestine-specific deletion of CGI-58 in CGI-58-floxed mice expressing villin promoter-driven Cre recombinase (CGI-58^f/f/cre^ mice), we performed immunoblotting (Fig. 1). As expected, the CGI-58 protein was abolished to undetectable level in the five equal segments of small intestine and it was also reduced dramatically in colon and rectum in CGI-58^f/f/cre^ mice. There were no changes of CGI-58 protein levels in other tissues including skin, heart, liver, muscle, and adipose tissues. These data demonstrate that the line of CGI-58^f/f/cre^ mice was indeed an intestine-specific CGI-58 knockout mouse model.

### Intestine-specific Inactivation of CGI-58 in Mice Causes Overaccumulation of TG in the Enterocytes of the Proximal Segments of Small Intestine

To examine whether intestinal CGI-58 deficiency causes LD deposition in enterocytes, we performed Oil-red O staining in the 4 h-fasted mice on regular chow diet (Fig. 1A). No intracellular LD accumulation was found in any segments of small intestine of CGI-58^f/f^ control mice. In CGI-58^f/f/cre^ mice, however, the enterocytic LD accumulation was severe in the first proximal segment of the 5 equal segments of the small intestine. The second proximal segment of small intestine from CGI-58^f/f/cre^ mice also showed LD accumulation. There was little or no LD accumulation in the enterocytes of the rest distal part of the small intestine of these animals. Similar pattern of LD deposition was observed for mice on HFD and in general males accumulated more enterocytic LDs than females (data not shown).

**Figure 2:**
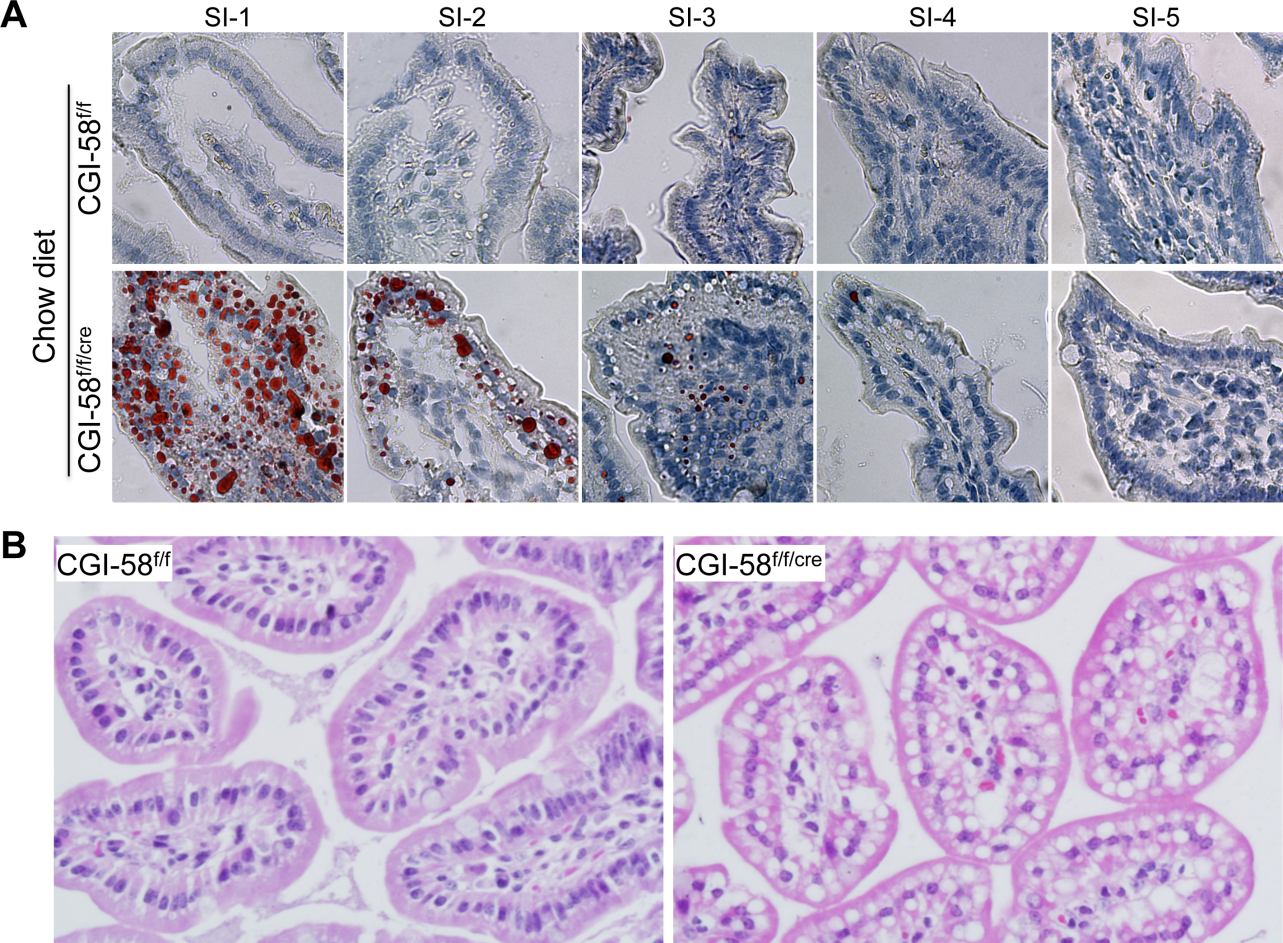
TG-rich LD accumulation in the cytoplasm of the proximal segment of small intestine of intestine-specific CGI-58 knockout mice. The entire small intestine was collected and separated into 5 equal segments (SI-1 to SI-5, proximal to distal). A: Oil-red O staining of the 5 equal segments of small intestine from 6-month-old male CGI-58^f/f^ and CGI-58^f/f/cre^ mice on chow diet. B: Hematoxilin & eosin (H&E) staining of the first proximal segment of small intestine sections from CGI-58^f/f^ and CGI-58^f/f/cre^ mice on HFD.

To examine the morphology of small intestine in our knockout mice, we conducted H&E staining of small intestine of mice on HFD for 6 weeks. No apparent differences were noticed for intestinal villus size and length between the two genotypes. Under high magnification, we saw many LD vacuoles in the cytoplasm of both apical and basolateral sides of enterocytes in the first proximal segment of small intestine from 4 h-fasted CGI-58^f/f/cre^ mice, but no such LDs were observed in the same intestinal segment of the CGI-58^f/f^ control mice (Fig. 2B).

**Figure 3:**
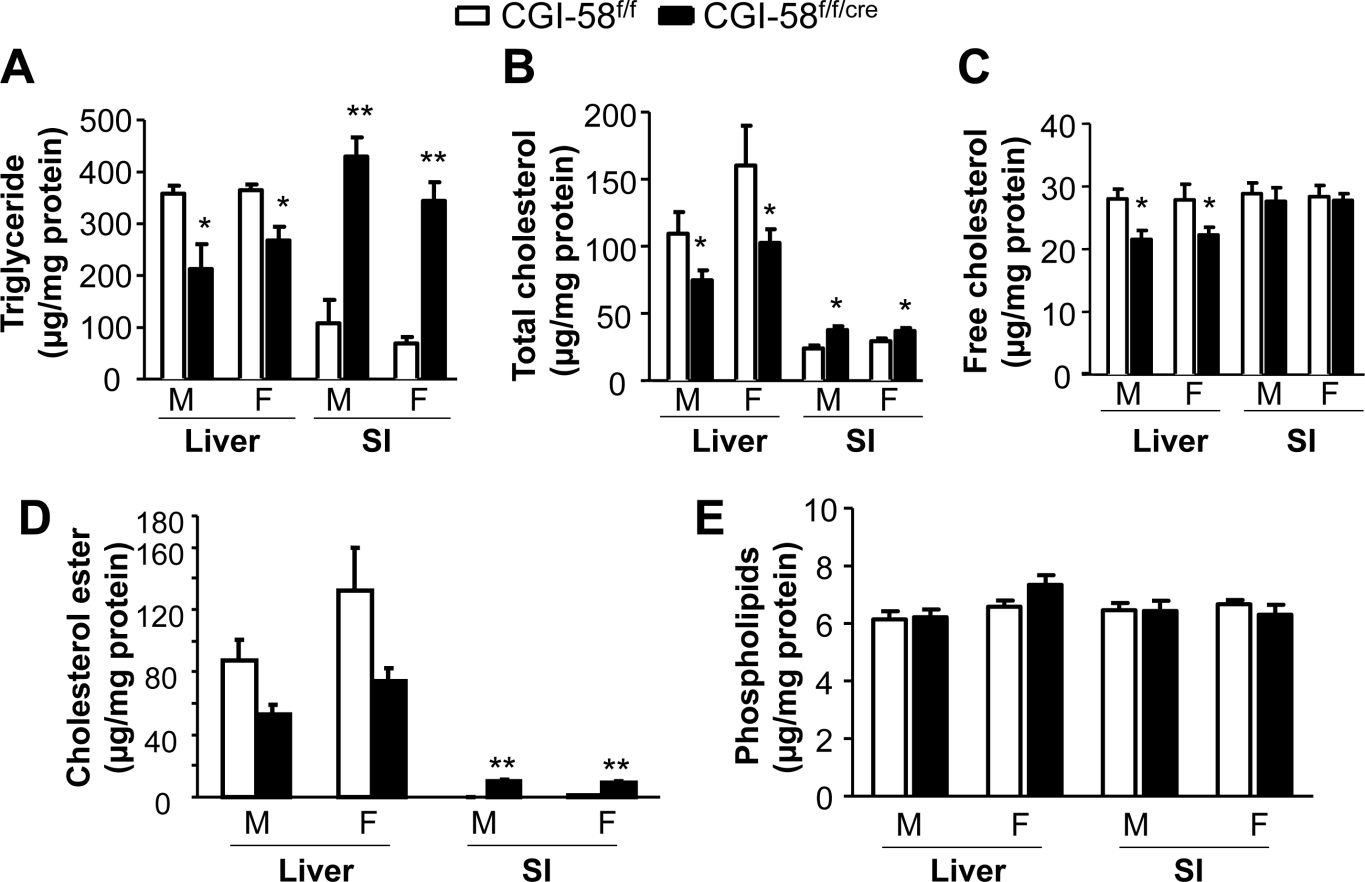
Lipid contents in the second proximal segment of small intestine (SI) and liver. Tissues were collected from CGI-58^f/f^ and CGI-58^f/f/cre^ mice on a Western-type diet for 6 weeks (n = 8), and tissue lipids were measured as described under *Experimental Procedures.* *P<0.05, **P<0.01 versus Control mice. M, male; F, female.

Biochemical quantification of lipid contents in the second segments of the small intestine (Fig. 3) shows that inactivation of CGI-58 in enterocytes of male mice increased TG content 4-fold (CGI-58^f/f/cre^ mice, 429.20±37.83 µg/mg protein vs. CGI-58^f/f^ mice, 108.11±44.94 µg/mg protein, *P*<0.01). The contents of intestinal total cholesterol and cholesterol ester, but not free cholesterol and phospholipids, were also significantly elevated in CGI-58^f/f/cre^ male mice. Similar changes in intestinal lipid contents were observed in CGI-58^f/f/cre^ female mice. On the contrary, the hepatic content of TG was decreased in the CGI-58^f/f/cre^ male mice compared to controls (CGI-58^f/f/cre^ mice, 212.7±47.8 µg/mg protein vs. CGI-58^f/f^ mice, 358.7±14.9 µg/mg protein). Both hepatic total cholesterol and free cholesterol contents were also significantly reduced in CGI-58^f/f/cre^ mice. There were no changes in liver PL content. In CGI-58^f/f/cre^ female mice, similar changes in hepatic lipid contents were also observed. The changes in hepatic lipid contents were not associated with alterations in liver and body weight (Table 1).

**Table 1:** Comparison of body weight, liver weight, and plasma parameters between control and intestine-specific CGI-58 knockout mice.

	Genotype and sex			
	CGI-58^f/f^ (Male)	CGI-58^f/f/cre^ (Male)	CGI-58^f/f^ (Female)	CGI-58^f/f/cre^ (Female)
BW (g)	26.7±1.2	25.8±0.7	20.7±0.7	21.8±0.5
Liver (g)	1.5±0.1	1.4±0.1	1.3±0.2	1.4±0.1
TG (mg/dl)	37.15±3.27	36.50±2.13	32.66±3.63	25.19±2.46
TC (mg/dl)	126.5±10.7	168.3±9.7*	92.7±2.6	102.8±3.3*
FC (mg/dl)	28.30±2.16	37.67±2.12**	20.46±0.55	22.92±0.93*
CE (mg/dl)	164.0±14.7	218.1±13.5*	120.6±3.8	133.4±4.3*
β-HB (mg/dl)	7.50±0.18	7.95±0.51	8.90±0.35	9.66±0.43
FFA (mmol/L)	0.64±0.03	0.68±0.03	0.64±0.03	0.72±0.02

Eight to ten mice were fasted 4 h and weighed to record the body weight (BW). The mice were sacrificed for collection of tissue and blood samples. The entire liver of each mouse was weighed. Plasma parameters were measured as described under *Experimental Procedures*. Data were analyzed by two-tailed Student’s *t* test between CGI-58^f/f^ (Control) and CGI-58^f/f/cre^ mice within the same sex.

*P<0.05,

**P<0.01. TC, total cholesterol; FC, free cholesterol; CE, cholesterol ester that was calculated by multiplying the mass difference between TC and FC by 1.67; β-HB, β-hydroxybutyrate.

### Increased Circulating Cholesterol in CGI-58^f/f/cre^ Mice

**Figure 4:**
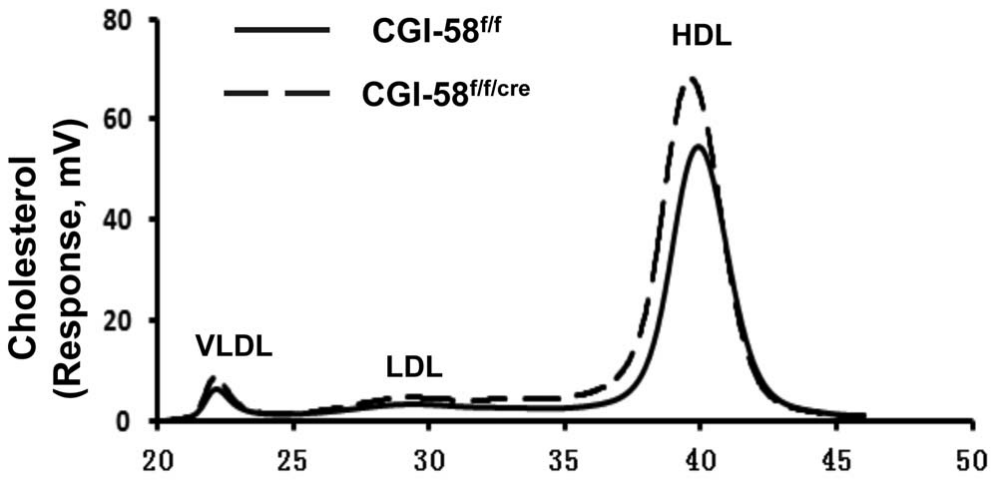
The plasma lipoprotein-cholesterol profile of pooled plasma samples. An equal amount (20 µl) of plasma sample from each male mouse in each group (n = 4) was pooled. The pooled sample was analyzed by FPLC as described under *Experimental Procedures*. VLDL, very low-density lipoprotein.

Table 1 also shows that there was an increase in plasma concentrations of total cholesterol, free cholesterol and cholesterol ester in CGI-58^f/f/cre^ mice. Plasma concentrations of TG, FFA and β-hydroxybutyrate remained unchanged in CGI-58^f/f/cre^ mice. Fast phase liquid chromatography (FPLC) analysis of the pooled plasma samples showed that the increased plasma cholesterol was seen among all lipoprotein particles (Fig. 4). Because the predominant lipoprotein particle in mice is the high-density lipoprotein (HDL), the increased cholesterol was mainly distributed in the HDL fraction. These data indicate that inhibition of CGI-58 in intestine altered the body’s cholesterol homeostasis.

### Impaired Fatty Acid Absorption in CGI-58^f/f/cre^ Mice

**Figure 5:**
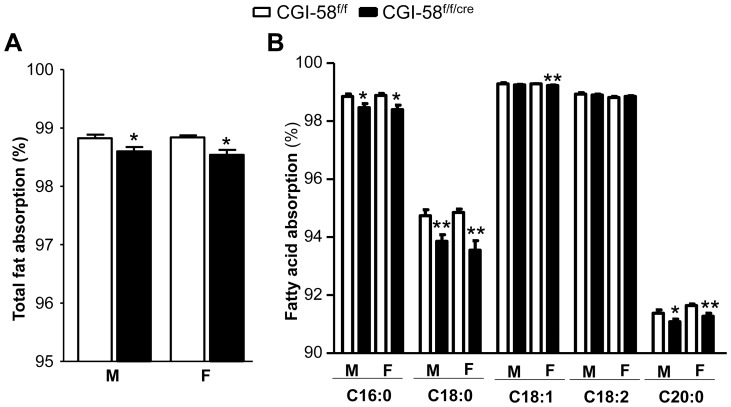
Intestinal fatty acid absorption in control males (n = 5) and females (n = 6) and in intestine-specific CGI-58 knockout males (n = 6) and females (n = 6). Mice at the age of 5 weeks were fed a HFD for 6 weeks, separated and individually housed, and then fed for 6 days a test diet including a fraction (5% of the total fat) of a non-absorbable marker sucrose poly-behenate (SPB). The fatty acid composition in both diet and feces, and the fractional absorption of total and individual fatty acids, were determined as described in *Experimental Procedures*. *P<0.05, **P<0.01 versus Control mice.

In general, intestinal fat absorption is very efficient. Using a very sensitive and accurate method with sucrose poly-behenate (SPB) as a non-absorbable marker [24], we compared the intestinal total fat absorption in CGI-58^f/f/cre^ and CGI-58^f/f^ mice and found that it was mildly but significantly reduced by 0.28% (*P* = 0.0176) in male and 0.31% (*P*<0.0117) in female CGI-58^f/f/cre^ (Fig. 5A). Slight but significant reductions were observed for palmitate (C16∶0), stearate (C18∶0), and arachidate (C20∶0) [0.47% (P = 0.0209), 1.10% (*P* = 0.0085), and 0.38% (*P* = 0.04) in males; 0.50% (*P* = 0.0156), 1.39% (*P* = 0.0034), and 0.4% (*P* = 0.0079) in females, respectively] (Fig. 5B). The intestinal absorption of oleate was significantly reduced by 0.06% (*P* = 0.009) in CGI-58^f/f/cre^ females, but no reduction was observed in CGI-58^f/f/cre^ males. There were also no changes in intestinal absorption of linoleate (C18∶2).

### Reduction of Postprandial TG Secretion in CGI-58^f/f/cre^ Mice

**Figure 6:**
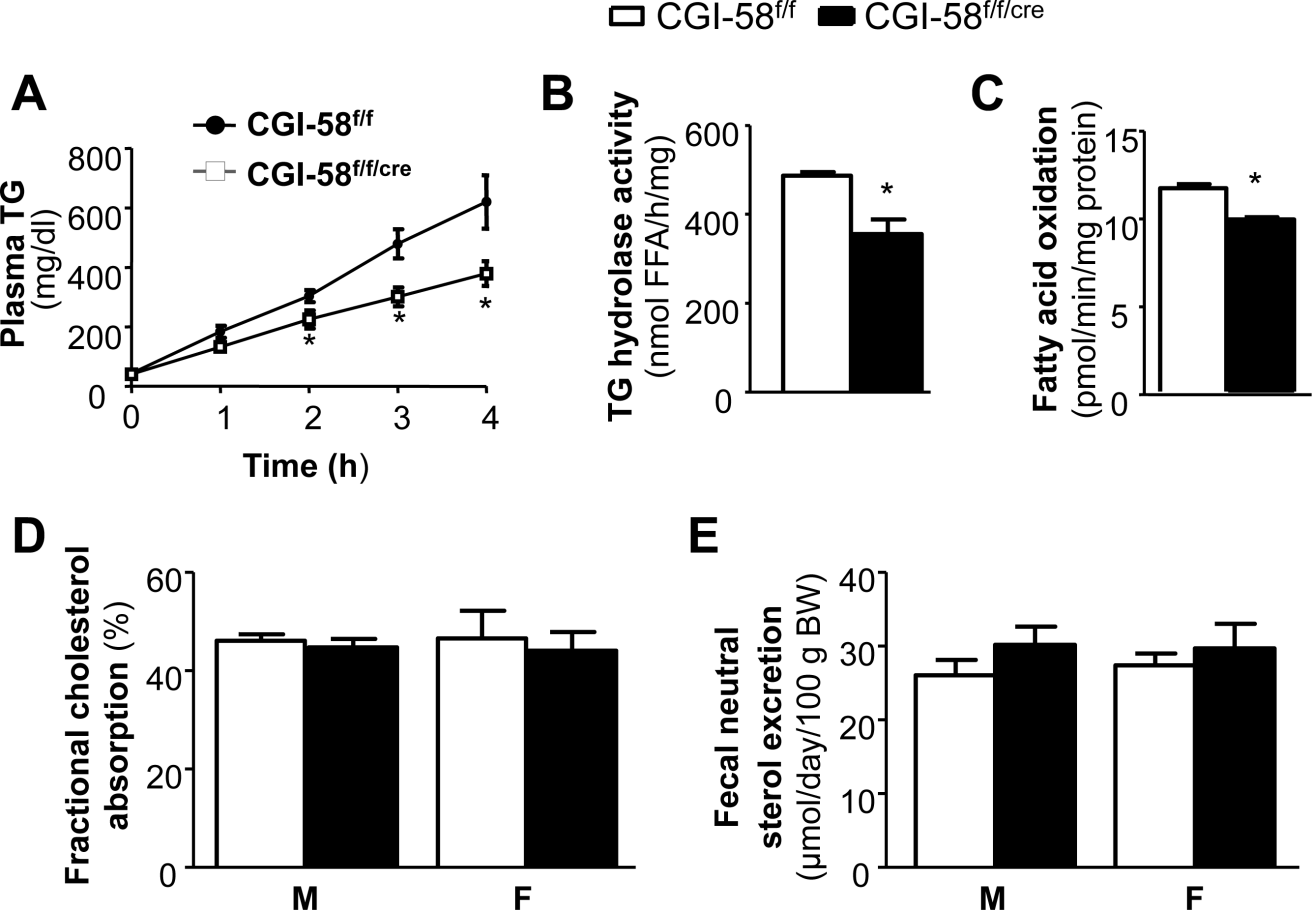
Decreased postprandial TG secretion, intestinal TG hydrolase activity, and intestinal fatty acid oxidation in intestine-specific CGI-58 knockout mice. A: Postprandial TG secretion. Male mice (n = 6) were pre-treated with the lipoprotein lipase inhibitor Tyloxapol (500 mg/kg) for 30 min and then administered by gavage of 0.5 ml of olive oil. Blood samples were collected at indicated times post oil administration, and analyzed for plasma TG concentrations. Measurements of TG hydrolase activity (B) (n = 6), rates of fatty acid oxidation (C) (n = 6), intestinal cholesterol absorption (D) (n = 8), and fecal total neutral sterol excretion (E) (n = 8) in male CGI-58^f/f^ and CGI-58^f/f/cre^ mice were performed as described under *Experimental Procedures*. *P<0.05 versus Control mice.

While CGI-58 deficiency in the intestine caused only mild, though significant, inhibitory effects on intestinal absorption of total fat and several free fatty acids over a 3-day period, we examined the effect of CGI-58 deletion on postprandial TG secretion into the blood stream. After overnight (16 h) fast, the mice were injected with a lipase inhibitor, followed by a bonus of 0.5 ml of olive oil and monitoring of plasma TG concentrations at different time points (Fig. 6A). The plasma TG contents were significantly lower in CGI-58^f/f/cre^ mice than CGI-58^f/f^ mice at time points 3 h and 4 h (301.7±32.1 mg/dl in CGI-58^f/f/cre^ mice vs. 479.2±48.9 mg/dl in CGI-58^f/f^ mice at 3 h; 379.5±41.3 mg/dl in CGI-58^f/f/cre^ mice vs. 620.3±90.2 mg/dl in CGI-58^f/f^ mice at 4 h, *P*<0.05) (Fig. 6A), suggesting a substantial reduction in postprandial lipoprotein-TG secretion in CGI-58^f/f/cre^ mice.

### Reduced Intestinal TG Hydrolase Activity and Fatty Acid Oxidation in CGI-58^f/f/cre^ Mice

As mentioned in the Introduction section, after a fatty meal, excessive TG may temporarily store in the cytosolic LDs and this pool of TG is eventually hydrolyzed for either re-esterification or assembly into CMs or for fatty acid oxidation. Given that CGI-58 enhances ATGL TG hydrolase activity in vitro, we determined if CGI-58 deletion influences intestinal TG hydrolase activity. As shown in Figure 6B, the TG hydrolase activity in the second proximal segment of small intestine was significantly reduced by 27% in CGI-58^f/f/cre^ mice (355.6±33.1 nmol FFA/h/mg protein in CGI-58^f/f/cre^ mice vs. 487.3±7.8 nmol FFA/h/mg protein in CGI-58^f/f^ mice, *P*<0.05). Reduced TG hydrolase activity could consequently reduce the supply of FFA substrates for oxidation. Indeed, we found that the rate of fatty acid oxidation was mildly yet significantly decreased in the second segment of small intestine from CGI-58^f/f/cre^ mice (11.66±0.23 pmol/min/mg protein in CGI-58^f/f/cre^ mice vs. 9.87±0.14 pmol/min/mg protein in CGI-58^f/f^ mice, *P*<0.05) (Fig. 6C).

### Cholesterol Absorption and Neutral Sterol Excretion in CGI-58^f/f/cre^ Mice

Given that cholesterol levels in intestine, liver and plasma were substantially altered in CGI-58^f/f/cre^ mice, we determined cholesterol balance by measuring intestinal cholesterol absorption and fecal neutral sterol excretion, and found that there were no differences between CGI-58^f/f/cre^ and control mice (Fig. 6D, E).

## Discussion

The major finding in this study is that inactivation of CGI-58 specifically in small intestine by Cre-mediated recombination leads to massive accumulation of TG-rich LDs in the enterocytes of the proximal part of small intestine (the major lipid absorption region of intestine) in fasted mice, recapitulating the original histopathologic findings in the small intestine of human CDS patients [8]. The LD accumulation in enterocytes is associated with a dramatic reduction in postprandial plasma TG concentrations, suggesting a reduction in postprandial lipoprotein-TG secretion. Mild, yet significant, decreases in intestinal absorption of total fat and some fatty acid species were also observed in these animals over a 3-day period. Since intestinal TG hydrolase activity is significantly reduced in CGI-58^f/f/cre^ mice, our data suggest that the lower postprandial plasma TG level is attributable, at least in part, to inhibition of mobilization of cytosolic LD-associated TG for lipoprotein-mediated secretion.

After a fat-rich meal, the quick formation of cytosolic LDs may have pathophysiological significance because it may alleviate FFA-induced lipotoxicity in a timely manner [26]. The postprandial formation of cytosolic LDs also indicates that the cellular machinery that transports fat out of the cell is saturable. Nonetheless, under normal circumstances, TGs in LDs of enterocytes are quickly hydrolyzed and then packaged into CMs for secretion. Some of released FFA may be channeled for oxidation locally as an energy source. The lipolytic factors promoting hydrolysis of cytosolic LD-associated TG in enterocytes are largely unknown. Our findings in intestine-specific CGI-58 knockout mice suggest that CGI-58 is a rate-limiting factor in mobilizing TG from cytosolic LDs for assembly into CMs for secretion. Consistently, in cultured hepatoma cells [27] and in the liver of antisense oligonucleotide (ASO)-treated mice [28], CGI-58-driven TG hydrolysis was shown to play an important role in mobilizing cytosolic TG for very low-density lipoprotein (VLDL)-TG secretion.

Dietary fat absorption is a highly efficient process and almost 95% of dietary fat consumed is absorbed [24], [29]. Mild, but significant, reduction in intestinal absorption of total fat and some fatty acid species (Fig. 5) due to the loss of CGI-58 is expected to lead to reduced weight gain over a long period of time. This would explain in part why most CDS patients are neither obese nor diabetic, despite fat deposition in non-adipose tissues (“ectopic” fat accumulation) [30], [31]. We found that the body weight was not different between CGI-58^f/f/cre^ and CGI-58^f/f^ mice on HFD for 6 weeks starting at 5 weeks of age (Table 1). However, it should be noted that the mice used in the current experiment were on the mixed genetic background, and future studies are required to systemically monitor weight gain in mice of pure genetic background.

The intestinal content of total, but not free, cholesterol was significantly increased in CGI-58^f/f/cre^ mice, indicating an increase in cholesterol esters. Considering enterocytic LD accumulation in CGI-58^f/f/cre^ mice, we speculate that cytosolic LDs may sequestrate cholesterol esters, thereby increasing its content in the intestine. Alternatively, CGI-58 may serve as a coactivator of a cholesterol esterase in the intestine, and in this case CGI-58 deficiency would expect to directly augment cholesterol ester content.

Interestingly, hepatic contents of TG, total cholesterol and free cholesterol were significantly reduced in the intestine-specific CGI-58 knockout mice (Fig. 3). Given that intestinal lipids were increased, it appears there was a redistribution of TG and cholesterol between intestine and liver in the CGI-58 knockout mice. Additionally, we found that plasma concentrations of total, esterified and free cholesterol were substantially elevated (Table 1). This elevation was not a result of alterations in intestinal cholesterol absorption and fecal cholesterol excretion (Fig. 6D, E). Detailed studies are required in the future to pinpoint the molecular mechanisms underlying this finding.

In summary, we created an intestine-specific CGI-58 knockout mouse model and using this model we demonstrated that intestinal CGI-58 is required for efficient postprandial lipoprotein-TG secretion and for maintaining hepatic and plasma lipid homeostasis. Our animal model will serve as a valuable tool to further define how intestinal fat metabolism influences the pathogenesis of metabolic disorders, such as obesity and type 2 diabetes.
